# B-Cell Acute Lymphoblastic Leukemia Presenting as Leukemia Cutis: A Case Report

**DOI:** 10.7759/cureus.11032

**Published:** 2020-10-19

**Authors:** Anoshia Afzal, Phillip Mingola, Umar Farooque, Sohaib Shabih, Cody A Thomas

**Affiliations:** 1 Pathology, University of Oklahoma Health Sciences Center, Oklahoma City, USA; 2 Neurology, Dow University of Health Sciences, Karachi, PAK; 3 Internal Medicine, Patel Hospital, Karachi, PAK

**Keywords:** leukemia cutis, b-cell acute lymphoblastic leukemia, humans, rare presentation, case report, b-all

## Abstract

Leukemia cutis (LC) is a manifestation of leukemia with infiltration of the dermis, epidermis, or subcutis by malignant leukocytes resulting in papules, plaques, nodules, or ulcers. It is usually associated with acute and chronic myeloid leukemia as well as T-cell acute lymphoblastic leukemia (T-ALL) but is very rare in patients with B-cell acute lymphoblastic leukemia (B-ALL). We report a case of a 58-year-old Hispanic male who presented with a non-healing leg ulcer of three months along with patches on the face, left arm, and bilateral legs with white blood cell (WBC) count of 50800/mm^3^ with 83% blasts, and flow cytometry findings of B-ALL. Punch biopsies from affected skin showed numerous dermal nodules composed of large atypical cells with open chromatin and prominent nucleoli. Immunohistochemical stains were consistent with B-ALL involving the skin and a diagnosis of LC was rendered. A high index of suspicion in relevant cases and prompt diagnosis is imperative to prevent any delays in appropriate therapy. Diagnosis in our case was aided by concurrent identification of B-ALL in the patient's peripheral blood. Since this information may not always be available, it is important to keep B-ALL in the differential any time there is a neoplastic infiltration of leukocytes in the dermis.

## Introduction

The infiltration of the dermis, epidermis, or subcutis by malignant leukocytes of leukemia can result in a cutaneous manifestation known as leukemia cutis (LC) [1]. It is generally related to acute, and chronic myeloid leukemia and T-cell acute lymphoblastic leukemia (T-ALL) but is very infrequent in patients with B-cell acute lymphoblastic leukemia (B-ALL). It is regarded as an aggressive manifestation of systemic leukemia [2]. The clinical picture of LC is variable and it can present as erythematous or hemorrhagic nodules, papules, plaques, ulcers, vesicles, or bullae of different shapes and sizes. The bullous lesions may show different morphologies and can appear on pre-existing cutaneous lesions or on normal skin [3]. Here we report the case of a patient with LC who presented with non-healing leg ulcer and multiple patches in various locations.

## Case presentation

**Figure 1:**
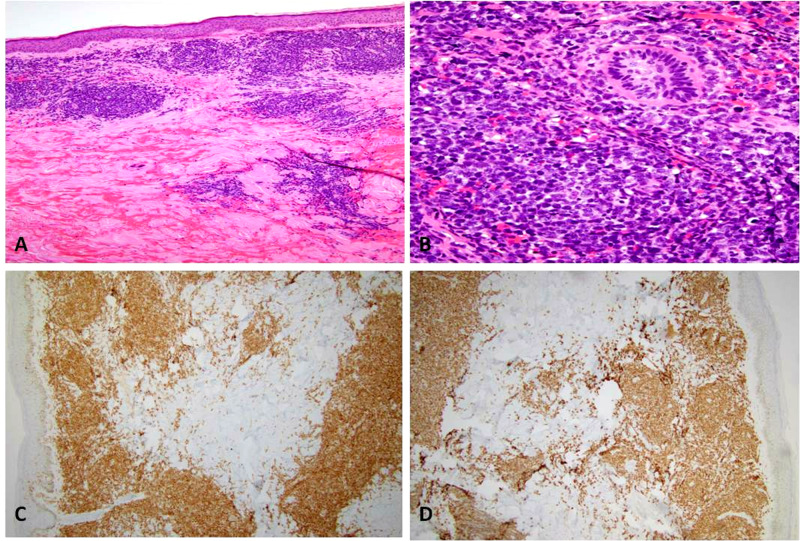
Hematoxylin and eosin staining shows multiple dermal nodules (A) composed of large atypical cells with open chromatin, prominent nucleoli, and multiple mitotic figures (B). Immunohistochemical staining reveals positive PAX5 (C) and TdT (D) in tumor nodules PAX: paired box; TdT: terminal deoxynucleotidyl transferase.

Our patient was a 58-year-old Hispanic male, with a previous history of hypertension, diabetes mellitus, and coronary artery disease, who presented for evaluation of a painless, non-healing leg ulcer of three months along with purple, well-defined patches on the face, left arm, and bilateral legs. Blood studies showed a white blood cell (WBC) count of 50800/mm3 with 83% blasts and flow cytometry confirmed B-ALL. Punch biopsies from involved skin revealed numerous dermal nodules composed of large atypical cells with open chromatin and prominent nucleoli (Figures 1A-1B). Immunohistochemical staining revealed the expression of paired box (PAX)5 and terminal deoxynucleotidyl transferase (TdT) which points towards B-cell lineage (Figures 1C-1D) [4,5].

**Figure 2:**
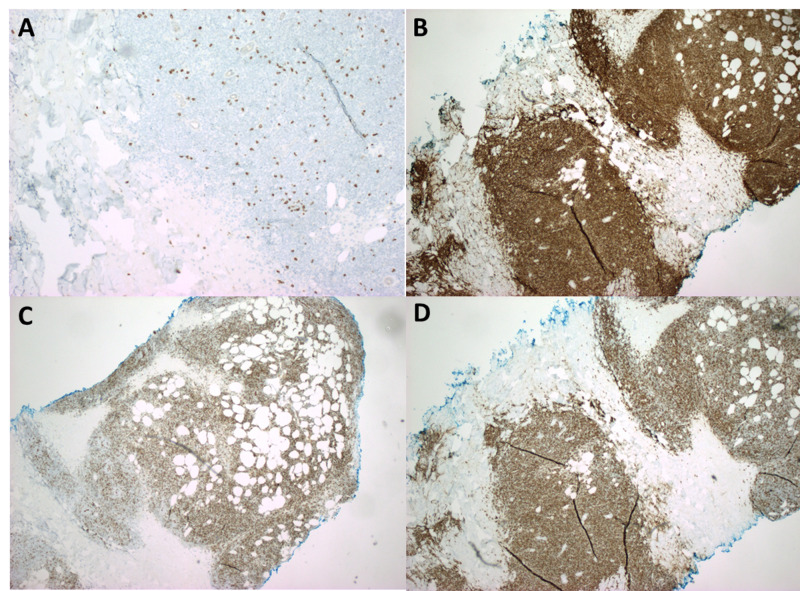
Immunohistochemical staining shows tumor cell population to be negative for CD3 (A) which rules out T-ALL; tumor cells are positive for CD10 (B) and CD20 (C) which are B-cell markers, and CD34 (D) which highlights the fact that these B cell clusters are lymphoblasts CD: cluster of differentiation; T-ALL: T-cell acute lymphoblastic leukemia.

The tumor nodules were negative for cluster of differentiation (CD)3 which ruled out T-ALL (Figure 2A). The tumor nodules were also positive for CD10, CD20, and CD34, further supporting the diagnosis of B-ALL involving the skin i.e. LC (Figures 2B-2D) [4,5].

The patient was started on appropriate chemotherapy as per the B-ALL protocol. He later developed septic shock due to bowel perforation and pancytopenia and expired within a few months of initial diagnosis.

## Discussion

There is very limited literature available that shows the association of LC with B-ALL. To our knowledge, only two cases of B-ALL associated LC have been reported previously. One patient presented with an asymptomatic, solitary, dome-shaped, indurated nodule on his left cheek in the absence of other systemic symptoms like fever, fatigue, weight loss, night sweats, or bruising/bleeding [6]. In the second case, a 46-year-old man presented with an erythematous, indurated, purplish nodule on his nose. He also had nodules on his forehead and forearm which were larger and erythematous [7]. Our case is unique since the patient presented with a non-healing leg ulcer and well-defined patches instead of erythematous nodules, which is otherwise the most common presentation in LC [8,9].

Based on the clinical presentation, acute/chronic myeloid leukemia and T-ALL were our major considerations as indicated by the available literature [10]. However, flow cytometry results, and immunohistochemical stain findings, showing neoplastic cells positive for PAX5, TdT, CD10, CD20, and CD34 and negative for CD3, confirmed the diagnosis of LC secondary to B-ALL [4,5].

B-ALL most frequently occurs in children, but it can be seen in adults as well. In addition to the reticuloendothelial system as the primary site of involvement, the central nervous system is the most frequent extramedullary site involved [11]. Although, the cutaneous infiltration is a common feature of T-ALL as compared to B-ALL on histopathological studies, the infiltrates in both T-ALL and B-ALL are deeply seated. The pattern of infiltration can vary from diffuse to perivascular. Countless mitotic figures, apoptotic changes, and stromal fibrosis are other prominent histopathologic findings [12]. Although cutaneous involvement is a rare occurrence in B-ALL, it depicts an advanced disease process and indicates a poor prognosis in adults as studies have shown a survival rate of 15% at six months after initial presentation [13].

## Conclusions

LC is an extremely rare finding in B-ALL, but it should always be considered in the differential diagnosis of rapidly progressing asymptomatic skin lesions (plaques, patches, nodules, or ulcers) with concomitant hematologic alterations. This can lead to an early diagnosis and initiation of appropriate treatment.
